# 非小细胞肺癌淋巴结结外侵犯的临床意义：研究现状与展望

**DOI:** 10.3779/j.issn.1009-3419.2026.101.03

**Published:** 2026-01-20

**Authors:** Han LIANG, Yajie MENG, Qi WANG, Caifan LI, Hao YANG, Xudong XIANG

**Affiliations:** ^1^650100 昆明，昆明医科大学第三附属医院，云南省肿瘤医院，北京大学肿瘤医院云南医院胸外二科（梁韩，王琪，李才繁， 杨昊，向旭东）; ^1^Department of Thoracic Surgery II; ^2^乳腺外科二病区（孟娅洁）; ^2^Second Ward, Department of Breast Surgery, The Third Affiliated Hospital of Kunming Medical University, Yunnan Cancer Hospital, Peking University Cancer Hospital Yunnan, Kunming 650100, China

**Keywords:** 肺肿瘤, 结外侵犯, 残留肿瘤, 预后, Lung neoplasms, Extranodal extension, Residual neoplasm, Prognosis

## Abstract

2022年全球癌症数据显示，肺癌位居全球癌症发病率及死亡率首位，而淋巴结转移是评估其预后及指导治疗的关键环节。淋巴结结外侵犯（extranodal extension, ENE）是指肿瘤细胞突破淋巴结被膜、侵犯周围组织的病理现象，在非小细胞肺癌（non-small cell lung cancer, NSCLC）中，尽管ENE尚未被正式纳入肿瘤原发灶-淋巴结-转移（tumor-node-metastasis, TNM）分期系统，但其临床价值已受到广泛关注。本文系统梳理ENE在NSCLC中的定义、诊断方法、预后意义及治疗相关研究进展，分析当前研究困境并提出未来展望。本文旨在为临床实践优化与后续研究开展提供参考。

## Abstract (en)

Global cancer statistics in 2022 ranked lung cancer as the leading cause of cancer incidence and mortality worldwide, with lymph node metastasis serving as a pivotal determinant of prognosis and treatment strategy. Extranodal extension (ENE) refers to the pathological phenomenon where tumor cells breach the lymph node capsule and invade surrounding tissues. In non-small cell lung cancer (NSCLC), although ENE has not yet been formally incorporated into the tumor-node-metastasis (TNM) staging system, its clinical value is gaining increasing attention. This review systematically summarizes the current research progress regarding the definition, diagnostic approaches, prognostic significance, and treatment implications of ENE in NSCLC, analyzes existing challenges, and proposes future directions. The goal of this paper is to provide insights for optimizing clinical practice and guiding subsequent research.

根据国际癌症研究机构（International Agency for Research on Cancer, IARC）发布的全球癌症数据^[1]^，2022年肺癌的发病率居全球首位，新发病例约248万例（占全球癌症总数的12.4%），同时也是癌症死亡的首要原因，对中国乃至全球公共卫生体系构成持续而严峻的挑战。在肺癌疾病进展过程中，淋巴结转移是评估预后和指导治疗的关键环节，其有无及严重程度直接影响疾病分期和治疗策略的选择^[2]^。研究^[3,4]^表明，相同病理N分期（pathological N staging, pN）的患者之间的预后仍具有异质性，而转移淋巴结负荷可能是造成这种差异的重要原因。因此淋巴结结外侵犯（extranodal extension, ENE）在非小细胞肺癌（non-small cell lung cancer, NSCLC）中的临床意义也日益受到关注。在头颈癌、乳腺癌等多种实体瘤中，ENE阳性已被证实是重要的不良预后因素，与更高的局部复发率和更差的生存结局有关^[5,6]^。尤其在头颈恶性肿瘤中，ENE因其突出的预后价值被纳入第八版美国癌症联合会（American Joint Committee on Cancer, AJCC）/国际抗癌联盟（Union for International Cancer Control, UICC）的肿瘤原发灶-淋巴结-转移（tumor-node-metastasis, TNM）分期系统中^[7]^，然而，在NSCLC领域，ENE的临床意义仍有诸多问题亟待阐明。

## 1 病理学定义及分级

### 1.1 ENE通用定义及其在头颈癌中分级

ENE亦称结外延伸或结外扩散，指转移癌突破淋巴结包膜进入结旁脂肪组织，这是多种癌症侵袭性的标志性特征^[8]^。最初，ENE的判定采用“存在”或“不存在”的二元分类。为了量化其严重程度，研究者将病理ENE（pathological ENE, pENE）区分为肉眼（宏观）ENE（肉眼可见的软组织受累）及显微镜下（微观）ENE（仅组织学上可见的病灶）^[9,10]^。尽管多项研究^[9,10]^显示肉眼pENE的预后较微观pENE更差，但二者的区分具有主观性和不精确性，因此这一结论并未成为共识。随后，Bhattacharya等^[11]^提出量化分级系统，将ENE系统划分为0级（肿瘤局限于淋巴结内）、1级（肿瘤达包膜且包膜增厚，但无突破）、2级（突破包膜≤1 mm）、3级（突破包膜>1 mm）、4级（无残余淋巴结结构，即软组织转移灶），但Lewis等^[12]^研究结果显示仅4级与较差预后有关。Prabhu^[13]^、Agarwal等^[14]^继续沿用这一分级系统，但常因任意设定的阈值而受到质疑。Yamada^[15]^、Woolgar等^[16]^尝试基于组织学的直观特征进行分型，但结果并未显示出明确意义，且这些分型判读仍具有较强的主观性。新近证据^[17,18]^表明，以2 mm为阈值具有预后意义，但这些单中心研究数据有限，尚需更大的多中心队列进行验证。基于既往研究，现行指南及头颈癌TNM分期系统并未在病理淋巴结分类中纳入ENE的确切范围，但建议记录扩展的程度为宏观或微观。宏观pENE定义为肉眼可见的结外扩展、显微镜下结外扩展>2 mm或显微镜下的软组织转移灶。微观pENE定义为显微镜下结外扩展≤2 mm^[19]^。同时，来自15个国家的19位病理学专家在国际头颈癌研究组（Head and Neck Cancer International Group, HNCIG）的共识^[20]^中以2 mm为关键阈值区分宏观及微观ENE，并且充分明确了软组织转移灶的定义。

### 1.2 ENE病理分级在NSCLC中的现状分析

尽管Lewis分级及HNCIG共识等标准为ENE的量化分级提供了重要参考，但这些分级体系的发展完善均基于头颈部恶性肿瘤，其关键阈值（如2 mm）是否适用于NSCLC尚不清楚。目前NSCLC相关研究中，ENE的判定常采用存在与否的二分类方法，并未对其浸润深度进行量化分级，目前仍没有研究对2 mm这一阈值进行预设并分组研究。这可能是由于肺门及纵隔淋巴结的解剖位置特殊、淋巴结切除途径的限制等导致NSCLC中ENE的病理评估难度远高于头颈癌。因此目前不应该盲目将这些分级系统应用到NSCLC中，但在病理报告中，应该详细记录ENE的有无及具体侵犯范围，为后续建立NSCLC特异性分级体系提供研究基础。

## 2 淋巴结ENE诊断

### 2.1 病理学诊断

目前，组织病理学是诊断ENE的金标准。但其对ENE的识别也面临诸多难题，首先，ENE的病理学定义以及显微镜下判读标准不统一。Abdel-Halim等^[21]^对44项研究的系统性综述显示，不同学者对ENE的定义描述高度不一致，这些定义大致分为3类，第1类（47.7%）：仅描述肿瘤突破淋巴结包膜（结节包膜破裂）；第2类（43.2%）：增加对周围组织的描述（需延伸至周围结缔组织）；第3类（9.1%）：进一步要求描述周围组织的特定反应（如需产生促纤维反应）。这些不一致性加剧了ENE病理学诊断难度。其次，诊断标准模糊以及判读专家主观性影响了ENE识别的准确性^[17,22]^，尤其在区分一些界限模糊的定义时（如包膜与假包膜）不同专家的阅读一致性较差^[12,21]^。最后，转移淋巴结本身的阅读具有复杂性，如术中淋巴结残缺、包膜不完整、淋巴结门部（不存在完整包膜）受累、促纤维反应干扰、脂肪组织干扰等^[17,21,22]^。针对上述问题，研究者尝试了多种改进策略：包括以“超越淋巴结轮廓”为诊断基准^[21]^、判读困难时通过其他征象辅助诊断（如促纤维反应、肿瘤与脂肪混合）^[17,22]^、淋巴结充分完整取材并且多层面切片^[12,22]^、影像学辅助^[23]^等。基于这些研究，HNCIG召集19位国际病理学家对ENE病理学定义以及诊断特征等达成共识^[20]^，共识规定淋巴结ENE统一术语为“extranodal extension (ENE)”，明确的诊断特征为肿瘤穿透淋巴结包膜全层或进入周围脂肪，共识还规范了ENE病理报告标准，对以往的诊断难点给出了解决指引。这些进展对肺癌的ENE诊断标准化具有重要启示。

### 2.2 影像学诊断

计算机断层扫描（computed tomography, CT）与磁共振成像（magnetic resonance imaging, MRI）是恶性肿瘤诊断最常用的影像学工具。其在检测ENE方面的价值已得到初步证实。多数研究均采用一种或多种临床ENE（clinical ENE, cENE）阳性判定特征，包括：淋巴结边界不清或轮廓不规则、周围脂肪间隙浸润、淋巴结融合或成簇，以及侵犯邻近结构（如血管、支气管）等^[24-31]^。其中MRI凭借其优异的软组织分辨率，以及不同加权序列的“火焰征”“毛刺征”等在评估淋巴结细微结构及周围浸润方面具有独特优势，但空间分辨率较低限制了其诊断的可靠性^[27,32]^。既往对MRI及CT诊断cENE的诊断性能报道参差不齐，根据meta分析^[32]^显示，CT和MRI诊断的敏感性分别为63%-77%和60%-85%，特异性分别为77%-85%和78%-96%。基于这些研究，HNCIG组织18位国际放射学专家进行了五轮改良德尔菲法讨论。将“淋巴结边缘或边界模糊或不规则”“延伸至结节周围脂肪”“向邻近结构（如肌肉、皮肤、腺体及神经血管束）的延伸”“淋巴结团聚、重叠和融合”确立为识别影像学检测到的ENE的标准。其中“淋巴结边缘或边界模糊或不规则”“延伸至结节周围脂肪”适用于CT及MRI，而“向邻近结构（如肌肉、皮肤、腺体及神经血管束）的延伸”“淋巴结团聚、重叠和融合”适用于MRI、CT及超声检查。同时，该组织还提出了cENE的标准化分级系统^[24]^：0级为无影像学可疑征象；1级表现为淋巴结边缘明显不规则或模糊，且延伸至周围脂肪组织但局限于该区域；2级为两个及以上不可分离的相邻结节（团聚/粘连/融合结节）的明确侵犯，伴或不伴1级特征；3级为出现对邻近结构（如肌肉、皮肤、神经血管或腺体）的明确侵犯，伴或不伴1或2级特征。该分级系统为cENE的标准化评估提供了重要框架。

正电子发射计算机断层显像（positron emission tomography/computed tomography, PET/CT）融合了形态学与肿瘤代谢信息，为ENE评估提供了新维度。研究表明，ENE阳性淋巴结的高代谢参数[如最大标准摄取值（maximum standardized uptake value, SUVmax）、肿瘤代谢体积（metabolic tumor volume, MTV）]是预测cENE的强有力指标^[33,34]^，但PET/CT同样受限于较低的空间分辨能力^[35]^。尽管现有报道有限，但多模态影像融合（如PET/CT、PET/MRI）可整合功能代谢（SUVmax、MTV）与形态学（如边缘毛刺）信息，展现出良好的应用前景^[36-38]^。超声在cENE诊断中的应用相对较少，其对cENE识别多基于CT/MRI特征，结合超声的回声强度进行判断^[39]^。Katayama等^[40]^研究显示，超声对cENE的诊断敏感性较高但特异性较低，可作为MRI的辅助工具。总体而言，各种影像学工具在cENE诊断中各有优劣，目前均无法对ENE做出完美识别。未来，传统影像学工具结合人工智能大模型、深度机器学习以及多模态融合或许是提高cENE诊断性能的关键^[36-38,41]^。

### 2.3 ENE诊断在NSCLC中的特异性挑战

目前，NSCLC中ENE的诊断多沿用头颈部恶性肿瘤的共识，但我们不应简单机械地应用。首先，与头颈部淋巴结不同的是，肺门及纵隔淋巴结长期暴露于空气污染及吸入性颗粒物中，常伴有显著的炭末沉着及纤维化改变。由此引发的慢性炎症及肉芽肿改变导致淋巴结包膜纤维化、扭曲或不完整，形态学上易表现为假阳性ENE^[42,43]^。同时，这种结构性变化影响淋巴正常引流，甚至干扰淋巴结的跳跃性转移模式^[44]^。在影像学层面，这些良性改变同样构成了巨大的诊断干扰：炭末沉着淋巴结中的肉芽肿反应常导致炎性细胞Glut-1酶水平升高，进而增加了^18^F-脱氧葡萄糖（fluorodeoxyglucose, FDG）的摄取（SUVmax>2.5）^[45,46]^。这种由良性疾病（如矽肺、肉芽肿）引起的高代谢状态，是导致PET/CT在评估肺癌淋巴结转移及ENE时敏感性高但特异性低的主要原因，极易造成误诊^[47,48]^。

此外，随着免疫检查点抑制剂（immune checkpoint inhibitors, ICIs）联合化疗成为局部晚期NSCLC的标准新辅助方案^[49]^，免疫治疗诱导的组织学与影像学改变进一步增加了ENE诊断难度。病理学方面，免疫介导的肿瘤退缩常表现为增殖性纤维化、新生血管形成及炎性浸润等“组织修复”征象^[50]^，因此新辅助治疗后，淋巴结包膜外出现的纤维化或炎症延伸至周围脂肪组织，将难以判定是ENE治疗后的瘢痕还是免疫治疗的特异改变，目前相关研究尚未对此做出明确界定^[43]^。影像学方面，免疫治疗可能引发特异性淋巴结免疫爆发反应，常表现为淋巴结体积增大或FDG摄取异常升高的假性进展^[51]^，这也使得影像学ENE判定更加困难。

因此，若评估者未能仔细区分这些良性反应，容易导致ENE的假阳性诊断，从而误导患者接受不必要的过度治疗。当前研究多局限于对现象的描述，未来研究则需要基于NSCLC特异性病理生理特征，建立适用于NSCLC的ENE诊断共识。

## 3 淋巴结ENE与NSCLC预后及复发转移

准确诊断ENE的最终目的在于评估患者预后并指导个体化治疗。下文将分别阐述组织病理学及影像学诊断ENE与NSCLC患者生存预后及复发模式的关系。

### 3.1 pENE与NSCLC预后

淋巴结转移状态是NSCLC的重要预后因素，但既往淋巴结分期仅基于病理淋巴结（pathological node, pN）的解剖位置及数量，难以解释相同N分期患者间的预后异质性^[52,53]^。研究发现，在pN分期的基础上，转移淋巴结负荷如不同转移模式（跳跃转移）^[4]^、阳性淋巴结比率（lymph node ratio, LNR）^[2,3,54,55]^以及ENE等均可以实现对患者预后更精确的分层，下文将重点阐述pENE与预后之间的关系。

#### 3.1.1 pENE与NSCLC预后分析

近年来，多项研究证实ENE与NSCLC较差的预后显著关联。Lee等^[56]^对199例患者的前瞻性研究显示，ENE阳性患者的5年生存率显著低于阴性组（13.5% vs 40.7%, P<0.001），并且II期ENE阳性患者预后差于IIIA期ENE阴性患者[5年总生存期（overall survival, OS）：16.8% vs 30.4%, P<0.001]，该研究首次对传统TNM分期发出挑战。Liu等^[41]^对388例患者的研究显示，ENE阳性是预后的独立预测因素，显著缩短了中位OS（30 vs 43个月）。Chen等^[57]^对279例肺腺癌的研究采用了国际肺癌研究协会（International Association for the Study of Lung Cancer, IASLC）建议的N分期亚组分类标准（将N1分为单站pN1a与多站pN1b），同样证实ENE阳性是生存预后的独立危险因素，并且该研究发现pN1b（ENE阳性）患者的生存劣于pN2a（ENE阴性）患者[无复发生存期（recurrence-free survival, RFS）与OS均P<0.001]，这一结果提示，传统的解剖学N分期可能低估了ENE阳性患者的风险。Ahn等^[58]^针对1713例患者的大样本研究进一步显示ENE是OS（HR=1.41, P<0.001）以及RFS（HR=1.49, P<0.001）独立预测因素，但该结论仅在腺癌亚组中有意义。Moon等^[59]^对375例肺腺癌的研究发现，转移性淋巴结大小（metastatic lymph node size, MLNS）>7 mm与ENE均为独立不良预后因素，两者并存时5年无病生存期（disease-free survival, DFS）最低（18.4%）。Nomura等^[60]^对168例肺腺癌的研究也证实，在淋巴结转移的各类形态学指标中，ENE是比肿瘤面积和坏死比例更重要的预后因素，并且后续结合人工智能辅助图像分析技术，对105例患者的研究再次证实ENE是NSCLC患者的独立预后因素^[61]^。

为了综合评估ENE的风险量级，Luchini等^[62]^纳入13项研究共1709例患者进行meta分析，结果显示ENE与全因死亡风险增加显著关联（合并RR=1.39，调整后HR=1.30）。另一项纳入5项研究（828例患者）的meta分析^[63]^也得出类似结论，ENE阳性状态会导致患者生存率显著降低（合并RR=0.45）。但上述meta分析均指出其结论受混杂因素影响。此外，Luchini等的研究^[62]^还揭示了既往研究间存在显著的异质性（I²=70%），从原始研究来看，尽管多数研究支持ENE是NSCLC的危险因素，但不同研究间的效应量差异较大（HR=1.233-3.291），这主要是因为各研究间ENE定义不统一，部分研究采用经典定义（突破包膜并侵犯周围纤维、脂肪组织）^[56,57,59,64,65]^，而部分研究将脂肪组织中的游离肿瘤细胞也纳入ENE^[41,60,66]^，这种定义上的差异可能导致了HR的波动，其次不同研究的设计差异，如研究的人群队列（部分仅为N1，部分为N1-N2，部分仅为N2）、切片方式（部分常规切片、部分连续切片）等也是HR波动的原因之一。

值得注意的是，Müller等^[64]^对118例pN2期NSCLC的研究得出了不同结论，其多因素分析显示ENE并非独立预后因素，这种结果的差异可能源于研究队列的特异性。首先，该研究群体中高达96.6%的患者接受了针对性辅助治疗（化疗/放疗/联合放化疗），而Yoon等^[66]^的研究表明辅助化疗可显著减轻ENE的负面影响（HR从1.58降至1.20），这解释了在规范化治疗队列中ENE负面影响的减弱现象。其次，Müller的研究对象为纯pN2期患者，多站淋巴结转移等强竞争风险因素可能掩盖了ENE的独立影响。相比之下，Shih等^[65]^对282例III-N2期患者的研究通过倾向评分匹配平衡了基线差异后，再次证实ENE是DFS的独立危险因素（HR=1.629）。

综上所述，ENE是NSCLC生存预后的独立危险因素。虽然ENE在各研究中显示的风险量级（HR值）参差不齐，但这主要源于各研究ENE定义、研究设计、治疗背景等的差异，同时，现有证据提示，ENE与MLNS、多站淋巴结转移等其他风险因素的结合可能比单一的ENE状态具有更好的预后分层价值^[59,64]^。

#### 3.1.2 复发转移模式

ENE阳性表现出复发模式上的复杂性，Liu等^[41]^研究显示，ENE阳性组3年局部及远处复发风险均显著高于阴性患者。而Chen^[57]^及Luchini等^[62]^研究中，ENE阳性仅显著增加整体局部复发风险，该结论在Lee^[67]^、Xie等^[68]^的大队列研究中也得到支持。Ahn等^[58]^采用竞争风险模型分析发现，ENE是局部复发独立危险因素[次分布风险比（subdistribution HR, SHR）=1.57, P=0.002]，但与远处复发无显著关联（P=0.090）。上述研究队列均为pN1-2期患者，未按N分期进行分层研究。与此不同，Yoon等^[66]^研究认为ENE仅显著影响远处复发（表1）。这些研究的差异可能与术中ENE阳性淋巴结清扫程度、ENE阳性淋巴结分布等因素有关，既往研究^[69,70]^显示，转移淋巴结的分布差异（如N1、N2、N1合并N2）是影响复发转移模式的重要因素，并影响术后辅助治疗的选择。因此，未来研究需要针对不同N分期进行分层分析，以更准确地评估ENE对NSCLC复发转移模式的影响。

**表1:** 近10年ENE与NSCLC预后相关性重要研究对比

Researcher	Year	Study population	N	n (%)	Research indicators	Recurrence pattern	HR (95%CI) (Multivariate analysis)	P
Liu, et al.^[41]^	2015	Stage pN1-2 Lung adenocarcinoma, squamous cell carcinoma	388	85 (21.9)	OS, DFS	Increased risk of both local and distant recurrence	OS: HR=1.233 DFS: HR=1.856	<0.05
Luchini, et al.^[62]^	2018	Stage pN1-2 lung adenocarcinoma, squamous cell carcinoma	1709	573 (33.5)	All-cause mortality	Increased risk of local recurrence	RR=1.39, HR=1.30	<0.05
Tabatabaei, et al.^[63]^	2018	Stage pN1-2 lung adenocarcinoma, squamous cell carcinoma	828	298 (35.9)	OS	-	RR=0.45	<0.00001
Moon, et al.^[59]^	2020	Stage pN1-2 lung adenocarcinoma	375	212 (56.5)	OS	-	OS: HR=1.454	<0.05
Chen, et al.^[57]^	2021	Stage pN1-2 lung adenocarcinoma	279	87 (31.2)	OS, RFS	Increased risk of local recurrence	OS: HR=3.291 RFS: HR=3.194	<0.001
Nomura, et al.^[60]^	2021	Stage pN1-2 lung adenocarcinoma	168	86 (51.2)	OS, RFS	-	OS: HR>2.0 RFS: HR>2.0	<0.05
Shih, et al.^[65]^	2021	pN2 stage lung adenocarcinoma, squamous cell carcinoma	282	85 (30.1)	DFS	-	DFS: HR=1.629	0.005
Müller, et al.^[64]^	2023	Stage pN2 lung adenocarcinoma and squamous cell carcinoma	118	51 (43.0)	OS, PFS	No increased risk of local recurrence	OS: NS PFS: NS	NS
Yoon, et al.^[66]^	2023	Stage pN1 lung adenocarcinoma and squamous cell carcinoma	862	-	OS, RFS	Inceased risk of distant metastasis	OS: HR=1.34 RFS: HR=1.39	≤0.05
Ahn, et al.^[58]^	2025	Stage pN1-2 lung adenocarcinoma, squamous cell carcinoma	1713	361 (21.1)	OS, DFS	Increased risk of local recurrence	OS: HR=1.41 DFS: HR=1.49	<0.001
Tsuchida, et al.^[61]^	2026	Stage pN1-2 lung adenocarcinoma	105	50 (47.6)	OS, RFS	Increased risk of both local and distant recurrence	OS: HR=2.23 RFS: HR=2.65	<0.05

-: The study did not describe the data; NSCLC: non-small cell lung cancer; ENE: extranodal extension; PFS: progression-free survival; pN: pathological N; OS: overall survival; DFS: disease-free survival; RFS: recurrence-free survival; NS: non significant; HR: hazard ratio; RR: relative risk.

#### 3.1.3 亚组分析

ENE的预后价值存在亚组异质性。组织学类型方面，Ahn等^[58]^研究提示ENE对OS和RFS的影响仅在腺癌患者中显著（HR=1.50-1.82）。这一发现在其余研究中也有报道，但目前缺乏针对非腺癌人群的研究，结论尚不能直接外推^[57,59,60]^。N分期方面，部分研究支持ENE在pN1和pN2患者中均为不良预后指标^[57,60]^，但也有研究显示ENE在不同N分期的预后价值存在差异，Luchini等^[62]^研究显示，ENE在pN1中的预后影响可能强于pN2（RR=1.63 vs 1.27）。Müller等^[64]^研究显示，pN2期NSCLC患者中ENE并未显示出良好的预后预测效果。

### 3.2 影像学诊断ENE与NSCLC预后

影像学诊断的cENE已超越单纯的诊断征象，成为头颈癌、直肠癌、前列腺癌、肺癌等的重要独立预后因素^[26,29,31]^，尤其在头颈癌中，cENE的预后价值已被纳入国际共识标准^[24]^。cENE的预后意义在于能够在治疗前无创性地识别出具有更高侵袭性生物学行为和更差临床结局的患者亚群。在NSCLC领域，Jang等^[26]^对382例cN1-2期患者进行了回顾性研究，这是该领域首个直接关联cENE与生存结局的大样本研究。结果显示，cENE是OS的独立危险因素（HR=1.72），并且患者的5年OS随cENE影像学表现程度的加重而显著降低（45% vs 21%-26%）。该研究的高特异性（87%-93%）提示，一旦影像学明确诊断ENE，便预示着较差的生存结局，但其局限性在于敏感性相对不足，导致部分潜在的高危患者被漏诊。相较于头颈癌，cENE对NSCLC的预后价值研究起步较晚，且面临更多挑战。目前，该领域的研究仍受限于缺乏统一的影像评估标准，这与纵隔淋巴结的结构复杂性及常规影像学对微小浸润识别能力有限有关。因此，需要更多基于高分辨率影像、多模态融合影像的大数据队列研究，以明确cENE在NSCLC 诊断-治疗-预后中的独立价值。

### 3.3 ENE与残留肿瘤分类

自1987年UICC引入残留肿瘤分类（residual classification，R分类）以来，该体系已被广泛用于评估肿瘤切除的完整性，并预测预后^[71]^。2005年，IASLC分期委员会提出新的R分类修订建议，其中一项重要提议是将ENE从R0重新分类为R1状态^[72,73]^。当时该提议因IASLC数据库中ENE数据有限（仅365例）而受到质疑^[68,73]^。然而，后续研究^[66,68,74]^逐步证实了该建议在pN1-2患者中的适用性。Yoon等^[66]^对862例pN1期患者的研究显示，在传统R分类标准下，R0-ENE阳性患者的预后显著差于R0-ENE阴性组（5年OS：51.6% vs 65.4%；5年RFS：44.4% vs 53.0%），仅与ENE阴性的R1/R2组相当，但这种差异在充分接受术后辅助化疗的人群中被抵消。Xie等^[68]^对4061例pN0-3期患者的大样本研究不仅支持IASLC将ENE升级为R1的提议，还发现ENE显著增加局部复发（如纵隔、同侧肺、恶性胸水）风险，这为“ENE代表不完全切除”提供了合理证据。该研究局限性在于无法校正辅助治疗异质性对生存预后的影响。Osarogiagbon^[74]^、Yun^[75]^及Oh等^[76]^针对pN2期患者的研究进一步验证了新IASLC R分类在该人群中的适用性。其中，Osarogiagbon等^[74]^基于北美多中心人群队列（3361例）的分析提供了较强的循证医学证据。虽然现有证据多源自回顾性研究，存在混杂因素及ENE评估标准不统一等问题，但这些结论依然是ENE组归为不完全切除的有力证据。

目前，ENE在肺癌TNM分期系统中的预后影响尚未完全明确，但早在2018年，IASLC的分期和预后因素委员会已将淋巴结结外侵犯的预后影响列为优先研究目标之一^[77]^。现有证据显示出ENE重要的临床提示价值，建议在病理报告中进行评估^[23]^，并将其作为pN分期分层的参考因素之一^[78]^。然而，在将其正式纳入分期系统前，仍需更多设计严谨的前瞻性、多中心研究加以验证。

## 4 ENE与NSCLC治疗选择

前文已对IASLC将ENE阳性患者纳入“不完全切除”范围的合理性进行论述，而这一改变将影响ENE患者的治疗管理策略^[67,76]^，下文将对ENE与治疗策略的联系进行论述。

ENE被定义为不完全切除（R1）及其所提示的肿瘤高侵袭性，为临床治疗策略的优化提供了理论依据。在术前，精准识别cENE有助于筛选适合新辅助治疗的人群，通过术前干预实现降期并清除微转移灶，从而提高R0切除率。目前，新辅助免疫联合化疗已在局部晚期NSCLC中显示出较高的病理缓解率^[49]^，但其对ENE患者的具体获益仍待探索。同时，在术中对pENE的准确评估是必要的，这强调了充分淋巴结清扫的重要性，也是准确评估ENE患者pN分期及淋巴结转移负荷的关键^[79,80]^。因此对于疑似ENE阳性的患者，更应完整清扫淋巴结并行病理检查，而非简单地取样活检^[67,75]^。

### 4.1 ENE与NSCLC辅助治疗选择

尽管术后放疗（postoperative radiotherapy, PORT）在乳腺癌、头颈癌伴ENE患者中的生存获益已被证实^[6,81,82]^，但在NSCLC中，对ENE阳性患者进行PORT的必要性长期存在争议。近期，Lung ART^[83]^及PORT-C^[84]^两项高级别研究显示，PORT并未改善完全切除后pN2期患者的DFS及OS，反而显著增加心肺毒性及相关死亡风险（Lung ART研究中，心肺相关死亡率：16.2% vs 2%）。然而，ENE阳性不仅意味着肿瘤的高侵袭性及复杂的复发转移模式，也代表着肿瘤的不完全切除，因此，这一特殊亚群不能简单依据单一因素进行PORT的决策，而需基于风险分层制定个体化策略。

现有证据表明，ENE阳性不仅是局部复发风险因素，也是全身微转移的强信号。Moretti^[23]^、Chien等^[80]^研究显示，PORT的选择仅改善了ENE阴性亚组的生存预后，而对ENE阳性亚组的生存预后改善并不显著。在这些研究中，ENE阳性与更高的远处转移风险显著关联，局部复发风险提高并不显著，而局部放疗无法控制全身进展。并且在Yoon等^[66]^的研究中，多因素分析显示辅助化疗可显著抵消ENE阳性带来的生存劣势（HR=0.49, P<0.05）。因此，无论N分期如何，强化的术后辅助全身治疗（含铂双药化疗、靶向治疗或免疫治疗）应作为ENE阳性患者的基础。

### 4.2 PORT在ENE阳性患者中的风险分层

Tabatabaei等^[63]^曾基于研究群体中ENE阳性局部风险增高的考量而建议考虑PORT，但该观点缺乏直接疗效数据支持。Borghetti等^[85]^也建议在pN1期非腺癌患者中，若ENE阳性和LNR>0.15患者可考虑PORT，然而研究未提供PORT疗效数据，其结论的可靠性值得质疑。既往高质量meta分析^[86]^显示，PORT并未显著改善pN0-1期患者OS，反而与放疗毒性导致的死亡风险升高有关（HR=1.21, 95%CI: 1.08-1.34, P=0.001）。目前，虽然不同研究对于ENE阳性患者复发转移模式有不同结论，但大部分研究并未对N分期进行分层分析，而Yoon等^[66]^的大样本数据提示，pN1 ENE阳性患者复发模式以远处转移为主（65.0%），Olszyna等^[87]^的研究中同样支持这一结论。同时，pN1淋巴结通常位于肺内或肺门，随肺叶切除一同移除，周围组织（如肺实质）通常已被切除，其造成肿瘤残留的风险低于纵隔pN2。因此，对于pN1 ENE阳性患者，术后治疗重心应以全身治疗为主，PORT的潜在危害大于获益，不应作为常规推荐，除非存在明确局部高危因素（如ENE阳性淋巴结紧邻支气管切缘）。

对于pN2患者，虽然Lung ART^[83]^和PORT-C^[84]^总体结果为阴性，但这些结果建立在完全切除（R0）基础上，研究并未否定PORT在特定高危亚组中的价值。而IASLC建议将ENE阳性视为“不完全切除”（R1）的生物学标志^[73,74]^，也为ENE阳性pN2期患者接受PORT提供了理论依据。此外，美国国立综合癌症网络（National Comprehensive Cancer Network, NCCN）指南也将PORT列为伴有ENE等高危因素的pN2期患者的可选方案^[80]^。因此，基于目前有限的证据，我们虽然不建议所有pN2 ENE阳性患者常规选择PORT，但对于多站N2转移^[54,88]^合并ENE阳性、高淋巴结负荷（如LNR>50%，阳性淋巴结数量>5等）合并ENE^[54,55]^，以及ENE阳性淋巴结与纵隔致密粘连或清扫不彻底、包膜外侵犯明显（肉眼可见或病理提示广泛浸润）等高局部复发风险人群，PORT是有必要的。而对于单纯、单站的pN2 ENE阳性患者，若已行彻底清扫且无上述叠加风险，全身治疗可能是更优先的选择，但需结合个体风险评估。

跳跃性转移pN2期疾病是一个特殊亚群，研究证实该亚群预后较好^[4,89]^，但既往对于pN2期是否选择PORT的研究中并没有对此亚群进行分层分析。虽然目前并没有研究直接证实ENE是否会抵消其预后上的优势，但Chen等^[57]^的研究中，ENE的存在显著改变了风险分层：pN1b（ENE阳性）患者的生存预后显著差于pN2a（ENE阴性）患者（RFS与OS均P<0.001）。基于此间接证据，相对合理的选择是，将跳跃性pN2合并ENE阳性群体按照非跳跃性pN2合并ENE阳性群体的标准进行风险评估。

## 5 总结与展望

ENE是多种恶性肿瘤的不良预后因素，在头颈恶性肿瘤中意义明确并被纳入TNM分期系统中。对NSCLC而言，ENE在TNM分期中的意义尚未明确，但现有研究已证实了ENE在NSCLC中的预后及治疗指导价值，并对传统pN分期提出挑战，未来研究应聚焦以下几个方向。

### 5.1 短期目标：构建NSCLC特异性标准化诊断共识

目前，NSCLC中ENE病理及影像学诊断多沿用头颈部肿瘤标准，缺乏NSCLC特异性的量化分级系统。首先，cENE是术前治疗规划的关键，但当前影像学诊断技术对pENE的诊断效能整体偏低，且对肺癌特异性良性疾病（如炭末沉着、肉芽肿结节等）及新辅助治疗等导致的慢性炎症及纤维化鉴别能力差，未来需要开展多中心影像学标准化研究，推动机器学习与影像组学发展，以及通过多模态影像结合（如代谢参数SUVmax/MTV联合CT/MRI纹理特征）提高cENE诊断性能及鉴别能力，并逐步建立影像学标准诊断共识。其次，病理学方面，目前存在的主要问题是，缺乏统一的评估标准及缺乏对ENE浸润程度的量化分级，同时，对肺癌特异性良性疾病及新辅助治疗等导致的组织学形态改变鉴别能力差，未来需要开展更多的多中心病理学研究，探索不同ENE浸润程度与NSCLC预后之间的关系，逐步建立统一的pENE诊断、鉴别标准及量化分级系统。

### 5.2 长期目标：探索ENE患者的个体化治疗方案及其与肿瘤微环境的关联

研究证实，ENE阳性提示了更高的潜在复发及转移风险，因此针对该群体的治疗需要更加精准的个体化风险分层，其中关键点之一是，验证术前cENE的精准识别并联合新辅助治疗诱导方案能否实现ENE转阴，达到真正的R0切除。另一个难点是在辅助治疗层面，如何针对性加强ENE阳性患者辅助治疗方案，以及如何根据患者风险分层进行PORT的合理选择，这些都缺乏相应循证医学证据，需要开展多中心、前瞻性的大队列研究来证实。其次，目前关于ENE与肿瘤微环境的研究缺乏，ENE的发生机制以及ENE与肿瘤微环境之间的相互作用尚不明确，寻找其发生分子机制及潜在干预靶点是未来亟待突破的关键点。
